# Aortic dissection at the University hospital of the West Indies: A 20-year clinicopathological study of autopsy cases

**DOI:** 10.1186/1756-0500-4-348

**Published:** 2011-09-09

**Authors:** Kathleen CM Coard

**Affiliations:** 1 Department of Pathology, The University of the West Indies, Mona, Kingston 7, Jamaica

## Abstract

**Background:**

An autopsy study of aortic dissection (AD) at our institution was previously reported. In the approximately 20 years since then, however, many aspects of diagnosis and treatment of this disease have changed, with a fall in mortality reported in many centers around the world. An impression amongst our pathologists that, there might be an increase in the prevalence of AD in the autopsy service at our hospital, since that earlier report, led to this repeated study, in an attempt to validate that notion. We also sought to identify any changes in clinicopathological features between the two series or any occurring during this study period itself.

**Findings:**

All cases of AD identified at autopsy, during the 20-year period since the conclusion of the last study, were collected and pertinent clinical and pathological data were analyzed and compared, both within the two decades of this study period and against the results of the last study.

Fifty-six cases comprised this study group including 36 males and 20 females, with a mean age of 63.9 years. There were, more patients in the second decade (n = 33; 59%) compared with the first decade (n = 23; 41%). Hypertension as a risk factor was identified in 52 (93%) cases and rupture occurred in 49 (88%) cases. A clinical diagnosis of AD was considered prior to surgery or autopsy in 25 (45%) cases overall, more during the second decade. Surgery was attempted in 25% of all cases with an increase in the second decade compared with the first.

**Conclusions:**

Compared with the earlier review, a variety of changes in the profile of patients with AD in the autopsy service has been noted, including a reversal in the female predominance seen previously. Other observations include an increase in cases where the correct clinical diagnosis was considered and in which surgical treatment was attempted, changes also evident when the second decade of the present study was compared with the earlier decade. Overall, there were many positive trends. However, areas that could still be improved include an increased index of suspicion for the diagnosis of AD and perhaps in the initiation of treatment, earlier, in those cases where the correct diagnosis was considered.

## Background

An appraisal of the frequency and pathological findings of aortic dissection (AD) in the autopsy service at the University Hospital of the West Indies (UHWI) was performed, for the first time > 20 years ago, in a study that spanned a 14-year period [1]. To-date, this is still the only such information available on patients with this disease in the English-speaking Caribbean. Much, however, about this condition has changed in the interim, including methods of diagnosis, management and prognosis. It was the author's impression that, in the years since the conclusion of the previous study, there had been an increase in the frequency of AD in the autopsy service. The objectives of this repeated study were therefore, to reexamine the cases of AD coming to autopsy in an attempt to validate that impression, to identify any clinicopathological changes occurring during the course of this extended survey and to compare the overall findings of this new study with that of the previous one.

## Methods

In a retrospective analysis, all cases of AD that presented to the autopsy service, during the 20-year period 1989-2008, were identified from our pathology department's post-mortem records. This represented a continuation of the period since the conclusion of the previous study. From these reports pertinent data were culled, including age and gender of patients and possible predisposing factors for AD including a history of hypertension. The diagnoses considered, by the clinicians, were also recorded and cases where surgery was attempted, were noted. The duration of hospital stay was also recorded, although only available in 24-hr increments. Pathological features noted from the autopsy included information regarding classification of the sub-type of dissection.

In this study, patients were classified as hypertensive if they demonstrated at least one of the following features: A history of hypertension, repeated blood pressure recordings of 160/90 mmHg or higher and autopsy evidence of cardiomegaly, defined as a heart weight > 350 g in males or > 300 g in females [2], in the absence of valvular disease or other heart disease known to be associated with increased heart weight.

For comparative analysis, data were divided into two 10-year periods representing the first and second decades of this study period. T-test and Chi-Square analyses were used to examine differences between the time periods as appropriate. The complete data were then compared with that from the earlier study.

## Results

During the 20-year period under review, 56 cases of AD were identified, with 33 (59%) patients in the second decade compared with 23 (41%) in the first decade (Table 1). In both decades, males outnumbered females. Altogether there were 36 males and 20 females, the majority occurring in the seventh and eighth decades of life (Table 2). The age-range of males was 39 to 93 years with a mean of 60.2 ± 12.9 years while that of females was 46 to 85 with a mean of 70.4 ± 11.9 years.

**Table 1:** Comparison of aortic dissection cases between the two decades

Parameters	1^st ^decade 1989-1998	2^nd ^decade 1999-2008	Both groups (20-yr period)
Number of cases	23 (41%)	33 (59%)	56
Gender ratio - M:F	16:7	20:13	36:20
			
Mean age (range) - M	65.2 (39-93)	56.3 (42-74)	60.2 (39-93)
Mean age (range) - F	68.9 (46-85)	71.2 (49-84)	70.4 (46-85)
Mean age overall	66.3 ± 14.9	62.2 ± 12.2	63.9 ± 13.4
			
Evidence of hypertension	20 (87%)	32 (97%)	52 (93%)
Mean heart weight - F ^a^	447.1 ± 160.4	427.1 ± 135.7	434.5 ± 141.2
Mean heart weight - M ^b^	457.2 ± 109.4	550.8 ± 131.6	509.2 ± 129.4
			
Diagnosis of AD considered	9 (39%)	16 (48%)	25 (45%)
Diagnosis of AD not considered	14 (61%)	17 (52%)	31 (55%)
			
Surgical Rx attempted*	2 (8.7%)	12 (36.4%)	14 (25%)
Ruptured cases (cases with attempted surgery)	22 (2)	27 (8)	49 (88%)

Rx = treatment

AD = aortic dissection

^a ^Normal range: 200-300 gm; mean 250 gm

^b ^Normal range: 250-350 gm; mean 300 gm

* p < 0.05; 1^st ^decade significantly different from 2^nd^

**Table 2:** Age and gender of patients with aortic dissection

	Male	Female	All
< 40	1	0	1
40-49	8	2	10
50-59	9	2	11
60-69	10	4	14
70-79	6	7	13
80-89	1	5	6
90 +	1	0	1

Total	36 (64%)	20 (36%)	56

Amongst the factors predisposing to dissection, 52 (93%) patients were adjudged to be hypertensive. Of these, only 3 patients (2 female) did not have cardiomegaly. There was a single recognized case of Marfan's syndrome, in a 42-year-old male. In one case, cross clamping of the aorta 6 hours earlier, at aneurysmectomy for an abdominal atherosclerotic aneurysm was adjudged to be the contributing factor. A predisposing factor for AD was not identified in 4 cases. A clinical diagnosis of AD was included, amongst the often multiple differential diagnoses proffered, in 25 of the 56 patients (45%), with an increase in the second decade (48%) when compared to the first (39%).

Forty-three (77%) dissections were Stanford type A and 13 (23%), type B (Table 3). Rupture of the dissection as recognized by hemorrhage, mostly into pericardial and/or pleural cavities, occurred in 49 (88%) cases, including 10 of the 14 patients who had been taken to surgery (Table 1). Of the 7 cases of AD that did not rupture, 2 died from sudden cardiac death due to extension of the dissection into a coronary artery. Both were clinically undiagnosed. Another died in congestive heart failure from aortic incompetence that, although recognized, was not attributed to the dissection with its accompanying annulo-aortic ectasia. The remaining 4 patients were surgically-treated patients who died from problems other than rupture. The mean heart weight of male patients deemed to be hypertensive was 509.2 ± 129.4 g (range 300-825 g) and that of female patients adjudged as hypertensive, 434.5 ± 141.2 g (range 295-760 g).

**Table 3:** Comparison of previous with the present study

Variables	Previous study	Present study
Duration	14 years	20 years
Number of aortic dissections	33	56
- Stanford type A	23 (70%) *	43 (77%)
- Stanford type B	10 (30%) *	13 (23%)
Mean age	62.4 yrs	63.9 yrs
Evidence of hypertension	27 (82%)	52 (93%)
Gender ratio	M:F 15:18	M:F 36:20
Correct diagnosis considered	5 (15%)	25 (45%)
Surgery attempted	0	14 (25%)
Ruptured cases	25 (76%)	49 (88%)

* Data changed from DeBakey to Stanford classification

Thirty-five patients died within the first day of hospital admission and 16 more within a week, most of these within the next 2 days. Of the remaining 5 patients, 4 were hospitalized for up to 2 weeks and one for 6 weeks. A diagnosis of AD was not considered in 3 of these latter cases including the patient who had the longest hospitalization. Surgery was ultimately performed in one of the remaining 2 cases but despite this attempt, it ruptured on the 8^th ^post-operative day. The diagnosis was only considered shortly before rupture in the last case.

Overall, surgery was attempted in 14 patients (9 with Stanford A and 5 with Stanford B dissections). There were statistically significant differences, between the two decades, in the number of cases where surgical treatment was undertaken, with more surgical attempts in the second decade (36.4%) compared with the first (8.7%). Ten of these ruptured, 8 during the early stages of the operation and 2 after surgery was completed, one 2 hours and the other 8 days later. The remaining 4 patients had a variety of surgical-related complications.

A comparison of the main findings of this 20-year study with those of the previous 14-year study is shown in Table 3. Among the differences seen was a reversal of the female predominance observed earlier. Also evident was an increase in the proportion of patients in whom a clinical diagnosis of AD was considered prior to surgery or autopsy. Compared to the previous study during which surgery was never undertaken, surgery was attempted in 25% of the patients overall, in this series. Efforts to identify surgically treated patients who had survived, led to confirmation of 8 patients within the last 11 years. However, these data are clearly incomplete.

## Discussion

Aortic dissection [AD] is generally regarded as infrequent but when it occurs, the consequences are often catastrophic. Although the more prevalent acute form is a life-threatening condition, with early mortality cited as high as 1 percent per hour among untreated patients [3], survival can be improved by the rapid introduction of appropriate medical and/or surgical therapy. Prompt diagnosis, therefore, remains essential if there is to be any real hope of successful management. Unfortunately, however, despite the fact that the diagnostic modalities and the treatment of this disease have greatly advanced in recent years, the mortality still remains very high in many parts of the world, to some extent because it is still under-diagnosed.

The results of this study suggest an increasing trend in the prevalence of AD at autopsy in the second decade compared with the first and in the overall prevalence of AD in this repeated study compared with the earlier one. Of course, these comparisons would only be valid if most patients with fatal AD came to autopsy. It is my belief that this should be the case and, at any rate, not different within the 2 study periods because of hospital policy regarding the indication for coroner's autopsies. These indications for coroner's autopsies include most patients who die within 24 hours of admission to hospital and within 24 hours of surgery, an interval embracing many of the cases of fatal AD. Local figures on autopsy rates have indicated that despite declining rates at our institution, as is the trend world-wide, the coroner autopsy rate has remained stable with even a marginal increase [4]. Others have also noted a similar trend of an increasing prevalence of AD at autopsy when compared with previous studies [5].

Despite debate about the etiology and pathogenesis of AD, there is no doubt that hypertension plays the most significant role, thought to be responsible for the initial intimal tearing [6]. It has even been suggested that, if systemic hypertension were eliminated, spontaneous AD would virtually disappear [7]. The apparent increased frequency of AD since the last study might be a reflection of the ongoing high prevalence of hypertension in Jamaica [8]. The high prevalence of hypertension in our study population was again supported by the magnitude of the mean heart weight of cases adjudged to be hypertensive, further validating the severity and duration in the cases under study. Unlike the findings of the previous study, there were no cases associated with pregnancy in this series and a single patient with Marfan's syndrome. However, a case deemed to be associated with cross clamping of the aorta at surgery, as occasionally described [9], was identified. Iatrogenic trauma as a predisposing factor for AD is said to be uncommon [10]. However, the possibility of a local increase in iatrogenic ADs exists as, with increased sophistication of services offered, even in developing countries like Jamaica, a growing number of patients is being subjected to cardiovascular surgery or invasive procedures of the aorta.

The mean age of patients in this series was similar to that of the previous local study, and similar to that of many other series including that of the International Registry of Acute Aortic Dissection [11]. An interesting difference between this and our previous study was a reversal of the highly unusual female predominance observed before [1]. Nearly all series report that AD occurs almost twice as commonly in men compared with women [11]. The male:female ratio of the present study thus conforms more to the well-recognized gender predisposition to this disease. The unusual gender bias in the previous study might have been a result of the small sample size.

Historically, the most widely quoted classification of AD was that of DeBakey [12] although, more recently, the Stanford classification [13] considered more useful from a treatment perspective, has increased in popularity. After transposing the findings from the DeBakey classification, used in the earlier study, to allow for comparison, the proportion of cases in each category was similar in both of our series, with a predominance of type A dissections. This ratio of type A to type B was slightly higher than that in the IRAD cohort [11].

Management of AD consists of aggressive antihypertensive treatment, and/or surgery. Optimal care of patients requires that the diagnosis be made promptly, including delineating the subtype of dissection, in order to facilitate planning for surgery for the Type-A variety. A range of modalities is now utilized to assist with diagnosis including computed tomography scanning, magnetic resonance imaging, and echocardiography, the choice depending on availability and expertise at different institutions [14]. Unlike the situation during much of the period covered in the previous study, these modalities are now available at the UHWI although admittedly, there are sometimes difficulties that result in inconsistent availability. Notwithstanding these limitations, however, it is apparent from this investigation that there could be a heightened index of suspicion, since a rapid diagnosis offers what little chance there is for an attempt at treatment of this highly fatal disease. While, compared to the previous study, there has been some improvement in the proportion of cases where the diagnosis of AD was considered, there were still more than half of the cases where this was not so. The major cause of the high mortality in a series from Hungary was attributed, by the authors, to failure of recognition and appropriate treatment of the acute dissections [15]. A similar high mortality of 93% was also reported in a small study from Puerto Rico [16]. This is in sharp contrast to the experience in some specialist centers, where there has been a significantly improved outcome within recent years [10]. In these institutions, while sophisticated diagnostic capabilities might play a major role, recognition of the disease promptly must also be the most important contributing factor to these favorable outcomes. In this regard, the improvement in diagnostic acumen in the second decade compared with the first is encouraging.

In contrast to that pertaining to the earlier survey period, we now have at the UHWI, the expertise and other facilities necessary for the surgical treatment of AD. Unfortunately, despite attempts to obtain such information form hospital records, the accurate mortality rate for surgically treated patients with AD at our hospital could not be obtained. Personal communication with the chief cardiovascular surgeon and information obtained from some records have, however, indicated continuing improvement in the survival of patients treated surgically. From our observations, one factor that might have contributed to death in some of these cases was the length of time between presentation and attempted surgery, an opinion supported by the observation that many of the deaths were due to rupture shortly after operative intervention was initiated. The relatively high proportion (one third) of type B dissections in our surgical population, might have added further to the mortality of the surgically-treated patients. Surgical intervention for acute type B dissections has been reserved for specific complications as it is well known that, the mortality following surgery for this type is consistently higher than with type A dissection [10,17,18].

There are some limitations to this study, most attributed to the fact that it is an autopsy study and a retrospective one. Recourse to clinical records might have included additional pertinent information particularly regarding more accurate timing of events and useful clinical data. However, efforts to obtain the hospital charts were mostly unsuccessful as these files were now inactive. Likewise, an attempt to obtain accurate information about the number of patients with successful surgery led to unreliable results. Despite these shortcomings, however, it is my opinion that this study provides useful follow-up information on an earlier study, indicating overall improvement.

## Conclusions

In summary, this repeated autopsy analysis has supported the suspicion that, compared to 20 years ago, there might be an increase in the prevalence of AD at the UHWI. It further revealed some clinicopathological changes between the first and second decades and other differences when compared to the earlier study including a reversal of the gender discrepancy noted previously, that might have been due to chance. There was evidence of progress in a number of the parameters examined, but there is room for continuing improvement particularly with regard to an increase in the clinical suspicion of AD and possibly in the initiation of treatment, in a more timely manner in those cases where this diagnosis was correctly considered.

## Competing interests

The author declares that she has no competing interests.
